# Demographics, clinical features and prognosis of patients with primary malignant conjunctival tumors at a tertiary hospital in Japan

**DOI:** 10.1007/s10384-025-01165-8

**Published:** 2025-02-17

**Authors:** Hiroshi Goto, Masaki Asakage, Erina Niidime, Naoyuki Yamakawa, Hiroyuki Komatsu, Kinya Tsubota, Kazuhiko Umazume, Yoshihiko Usui, Hideki Mori

**Affiliations:** Department of Ophthalmology, Tokyo Medical University, 6-7-1 Nishi-shinjuku, Shinjuku-ku, Tokyo, 160-0023 Japan

**Keywords:** Malignant conjunctival tumor, Lymphoma, Squamous cell carcinoma, OSSN, Melanoma

## Abstract

**Purpose:**

To clarify the demographic characteristics, clinical features and prognosis of patients with primary malignant conjunctival tumors diagnosed at a single tertiary hospital in Japan.

**Study design:**

Retrospective, observational case series.

**Methods:**

Patients with malignant conjunctival tumors diagnosed histopathologically at Tokyo Medical University Hospital between 2010 and 2022 were retrospectively reviewed. The demographic profile, clinical features and treatment outcomes were analyzed.

**Results:**

A total of 359 patients with histopathologically proven malignant conjunctival tumors were included. All patients were Japanese. The most common malignant conjunctival tumor was lymphoma (*n* = 197, 54.9%), followed by squamous cell carcinoma (SCC) (*n* = 97, 27.0%), melanoma (*n* = 60, 16.7%), and others (*n* = 5, 1.4%). The mean age of patients at the time of diagnosis was 60.5 ± 17.4 years for lymphoma, 60.2 ± 15.8 years for SCC, and 65.8 ± 15.7 years for melanoma. Among 197 patients with lymphoma, 89.3% had extranodal marginal zone lymphoma (EMZL). Lymphomas were treated with external beam radiation therapy in 52.8%, surgical resection with or without cryopexy in 40.1%, and systemic chemotherapy in 4.0%. During an observation period of more than 12 months, recurrence was observed in 8.4% and extraocular lymphoma occurred in 1.7%. In SCC and melanoma, common surgical resection with sufficient safety margin, cryopexy, and application of 0.04% mitomycin C were performed as eye-preserving therapy. Orbital exenteration was performed in advanced cases. Recurrence rates and regional lymph node metastasis rates during an observation period of more than 12 months were, respectively, 30.1% and 6.2% in SCC. Recurrence rates and regional lymph node or distant organ metastasis rates during an observation period of more than 24 months for melanoma were 29% for both. Tumor-related mortality was 16% in melanoma, but 0% in lymphoma and SCC.

**Conclusions:**

Lymphoma was the major malignant conjunctival tumor in a Japanese ophthalmology referral center, which may reflect a unique epidemiological trend in Japan compared to Western countries. The prognosis of lymphoma and SCC after appropriate treatment was favorable, except for some cases of advanced SCC.

## Introduction

Primary malignant tumors arising from the conjunctiva are relatively rare. Although there are many publications on ocular surface squamous neoplasia (OSSN) and conjunctival melanoma from Europe and the United States [1–6], reports from Asia, especially from Japan are limited [7, 8]. Additionally, there may be ethnic differences in the frequencies of conjunctival malignant tumors; for example, sebaceous carcinoma of the eyelid is much more common in Asia including Japan than in Western countries [9–12]. However, few reports address such potential epidemiological differences in malignant conjunctival tumors.

The purpose of this study was to investigate the demographics, clinical features, and treatment outcomes of primary malignant conjunctival tumors diagnosed in a single ophthalmology referral center in Japan since the past decade.

## Methods

Data from patients with primary malignant conjunctival tumors diagnosed histopathologically at Tokyo Medical University Hospital between 2010 and 2022 were retrospectively reviewed. Hematoxylin-eosin staining and immunohistochemical staining were performed as needed for the histopathological diagnosis, using samples obtained by incisional biopsy or excisional biopsy. Flow cytometry and detection of rearrangement of immunoglobulin heavy chain gene (JH region) by Southern blot were conducted for the diagnosis and classification of lymphoma, according to the WHO classification system [13].

The numbers of patient diagnosed with different tumor types during the study period, demographics including age and sex, and tumor laterality at the time of diagnosis were reviewed.

Secondary conjunctival lymphoma that apparently developed from adjacent ocular adnexa or during the course of a known systemic lymphoma were excluded. Among OSSN, dysplasia and conjunctival intraepithelial neoplasia (CIN) were excluded from this study and only squamous cell carcinoma (SCC) was investigated. SCC involving the lacrimal duct was excluded, because of unclear original site.

Positron emission-computed tomography (PET-CT), enhanced CT or magnetic resonance imaging (MRI) were performed as needed to detect extraocular lesions and/or metastasis during the follow-up period.

Student’s *t* test and Chi-square test were used for statistical analysis. A *P* value less than 0.05 was considered statistically significant.

This study was approved by the institutional review board of Tokyo Medical University (Approval Number: SH3281). Informed consent was waived for this retrospective study following local ethical guidelines.

## Results

A total of 359 patients with histopathologically diagnosed malignant conjunctival tumors were included in this study. All patients were Japanese. The mean age of all patients at the time of diagnosis was 61.5 ± 16.8 years. There were 162 men (45.1%) and 197 women (54.9%). Forty patients (10.6%) had bilateral conjunctival lesion.

Table 1 shows the number of patients, mean age, sex ratio, and tumor laterality of different tumor types. The most common tumor was lymphoma (*n* = 197, 54.9%), followed by SCC (*n* = 97, 27.0%), melanoma (*n* = 60, 16.7%), basal cell carcinoma (BCC) (*n* = 2, 0.6%), Kaposi’s sarcoma (*n* = 2, 0.6%), and solitary fibrous tumor (SFT) (*n* = 1, 0.3%). The top three diseases; lymphoma, SCC, and melanoma, totaled 354 cases (98.6%).

**Table 1:** Numbers of patients and demographic profile of malignant conjunctival tumors

	Number of patients (%)	Age (years) Mean ± S.D. (range)	Sex ratio Men:women	Tumor laterality Right:left:bilateral
Lymphoma	197 (54.9)	60.5 ± 17.4 (18–101)	77:120	79:83:35
Squamous cell carcinoma	97 (27.0)	60.2 ± 15.8 (27–95)	54:43	43:49:5
Melanoma	60 (16.7)	65.8 ± 15.7 (28–96)	28: 32	32:28 :0
Basal cell carcinoma	2 (0.6)	76, 52	1:1	0:2 :0
Kaposi sarcoma	2 (0.6)	44, 28	2:0	0:2:0
Solitary fibrous tumor	1 (0.3)	35	0:1	0:1:0
Total	359 (100)	61.5 ± 16.8 (18–101)	162:197	154:165:40

**Fig. 1:**
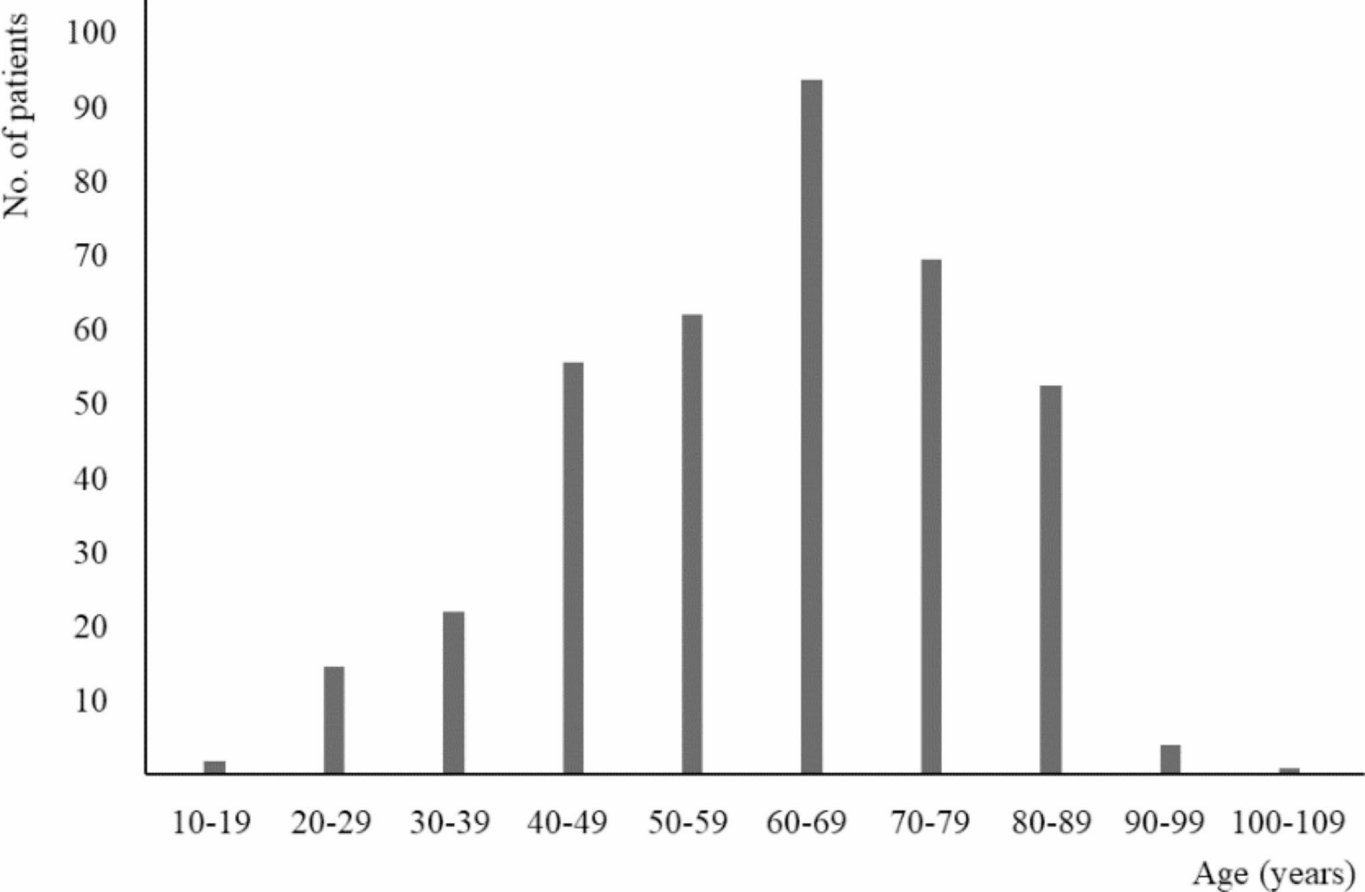
Age distribution of all patients with primary malignant conjunctival tumors

Figure 1 shows the age distribution of all patients. The peak age of patients diagnosed with primary conjunctival malignant tumors was in the 60s. The youngest patient was an 18-year-old woman with lymphoma, and the oldest patient was a 101-year-old man with lymphoma. There were no significant differences in age between the three major malignant conjunctival tumors (lymphoma, SCC, and melanoma, *t* test).

Detailed clinical data and treatment outcomes of each tumor are described below.

### Lymphoma (*n* = 197)

The mean age at the time of diagnosis of lymphoma was 60.5 ± 17.4 (range: 18–101) years. There were 77 men (39.1%) and 120 women (60.9%), and the proportion of women was significantly higher than of men (*P* = 0.0012, Chi-square test). Bilateral disease was observed in 35 patients (17.8%). Histopathologically, extranodal marginal zone lymphoma (EMZL) was the most prevalent type (176 patients, 89.3%), followed by follicular lymphoma (14 patients, 7.1%), mantle cell lymphoma (3 patients, 1.5%), diffuse large B cell lymphoma (2 patients, 1.0%), and T cell lymphoma (2 patients, 1.0%). Systemic work up at the time of diagnosis confirmed the presence of extraocular lymphoma in 19 cases (9.6%).

**Fig. 2:**
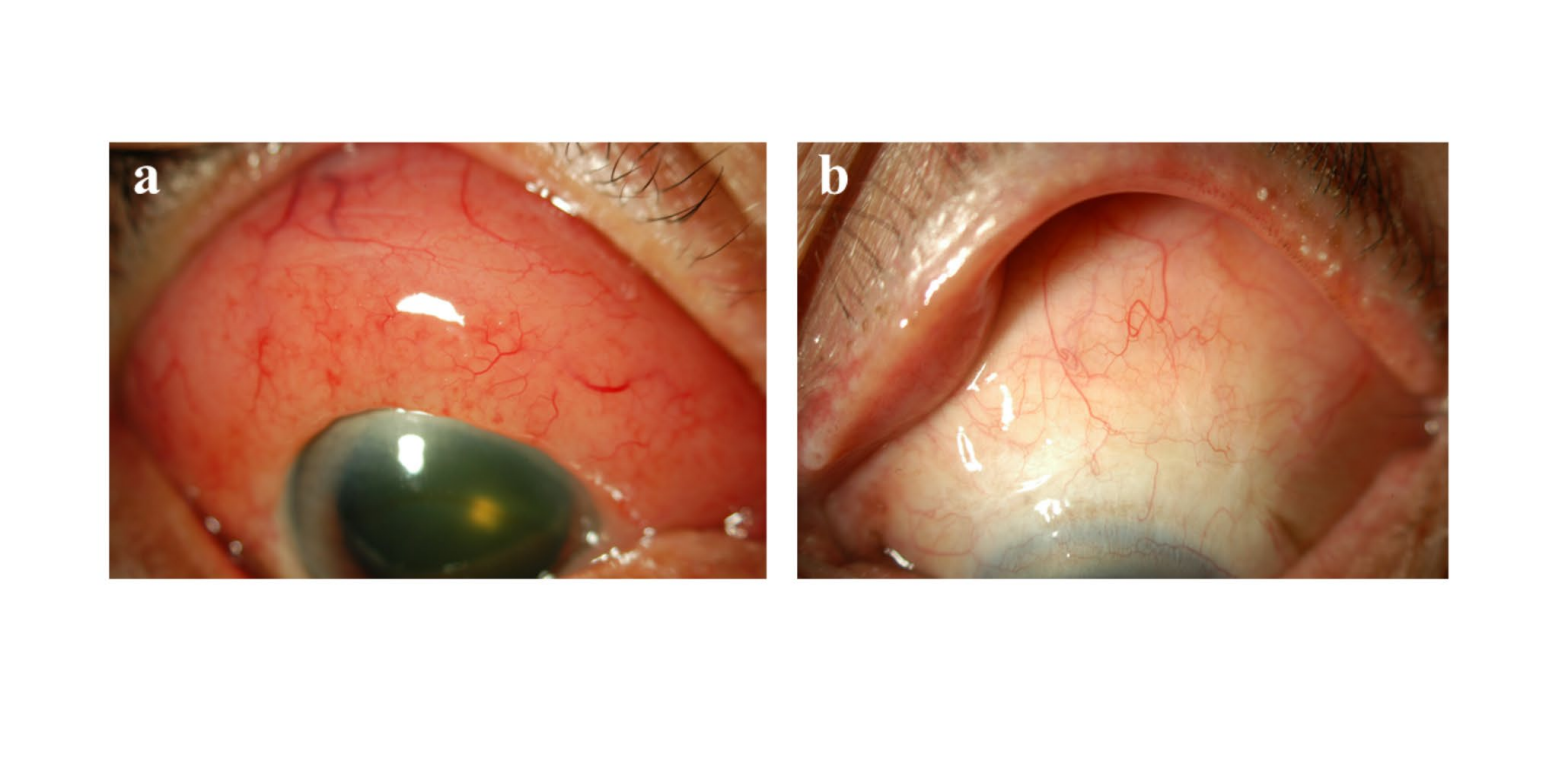
Conjunctival lymphoma: before treatment (**a**) and after treatment with 20 Gy of electron beams (**b**)

**Fig. 3:**
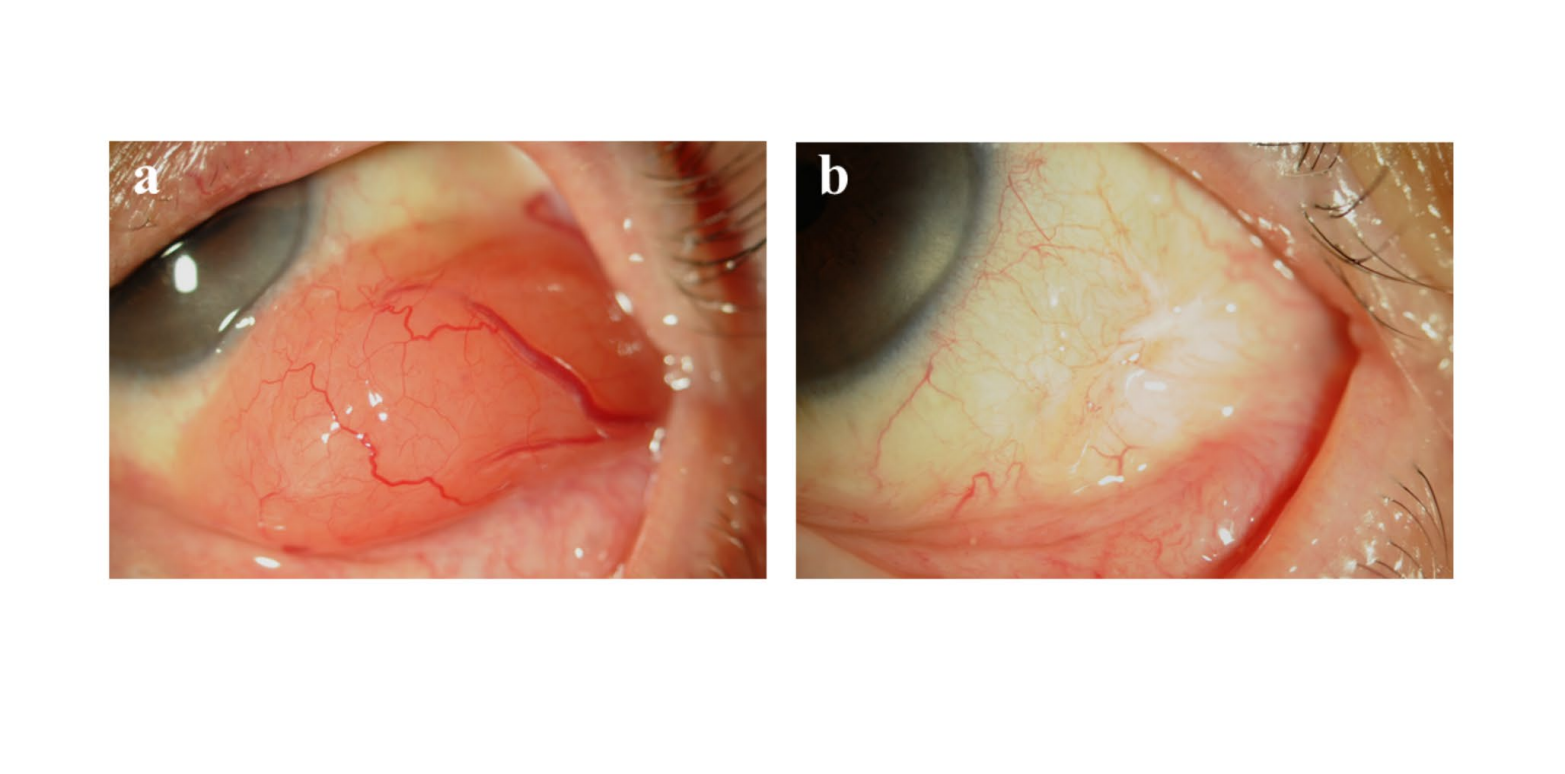
Conjunctival lymphoma: before treatment (**a**) and after extraction of the tumor located underneath the bulbar conjunctiva (**a**). No recurrence was observed for 24 months

Among 197 patients, 104 (52.8%) were treated with external beam radiation therapy (EBRT) at a dose of 20–30 Gy using electron beams (Fig. 2), 79 (40.1%) were treated with surgical resection with or without cryopexy (Fig. 3), 7 were treated with systemic chemotherapy, and 1 was treated with EBRT and systemic chemotherapy. The remaining 6 patients were treated at other hospitals or were lost to follow-up.

Treatment outcomes of 178 patients followed for 12 months or longer at our hospital were analyzed. The mean follow-up period was 28.3 ± 22.4 months. Local recurrence was observed in 15 patients (8.4%), and novel extraocular lymphoma was confirmed in 4 patients (1.7%) after treatment. None of the 178 patients died from lymphoma during the follow-up period, although the exact mortality status is unknown, as a few patients were transferred to other hospitals when systemic lymphoma was confirmed.

### Squamous cell carcinoma (*n* = 97)

The mean age of patients when diagnosed with SCC was 60.2 ± 15.8 (range: 27–95) years. There were 54 men and 43 women, with no significant difference in proportions of men and women. Bilateral lesion was observed in 5 patients (5.2%). Thirteen patients (13.4%) had a medical history of atopic dermatitis.

**Fig. 4:**
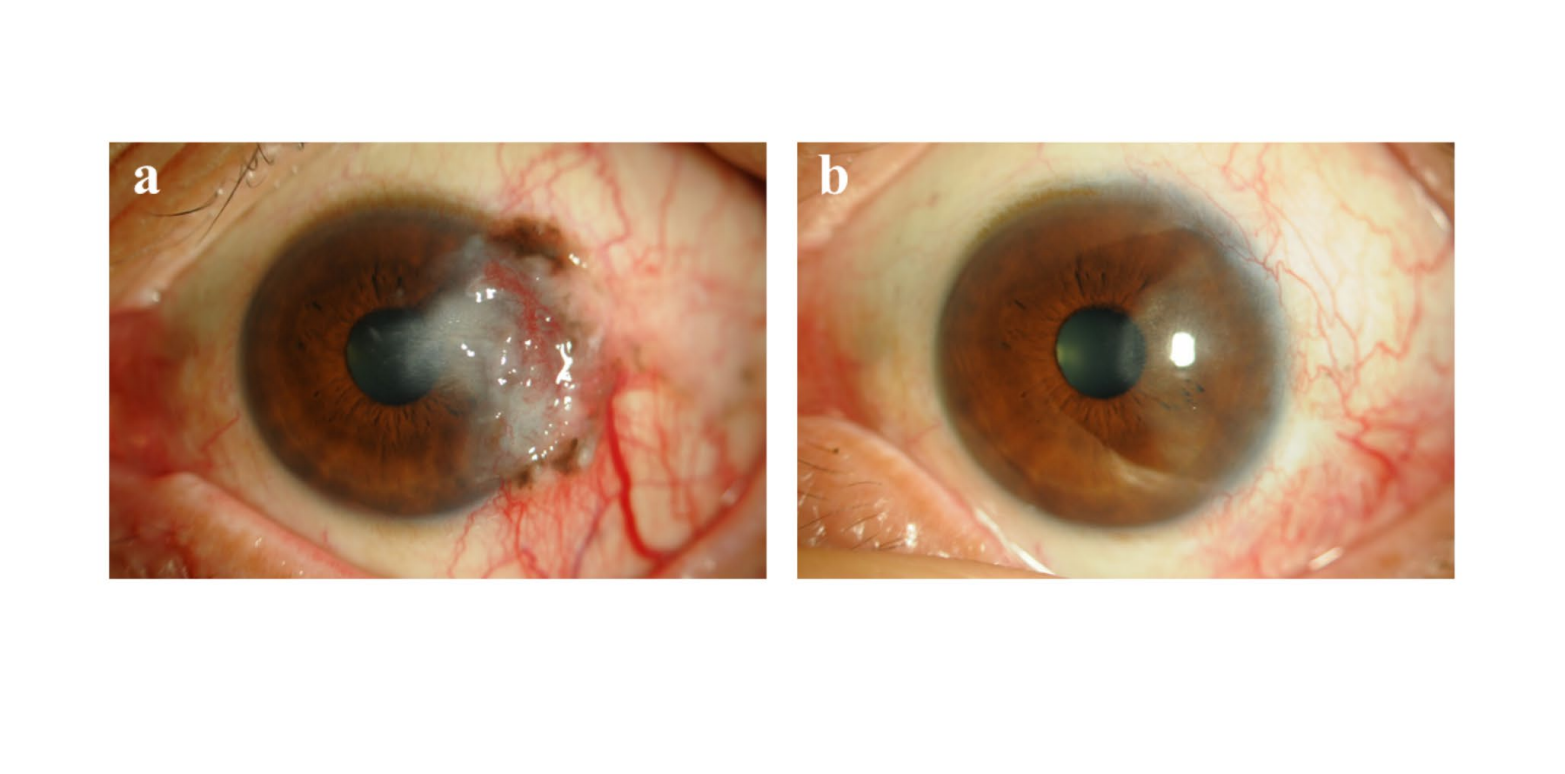
Squamous cell carcinoma: before treatment (**a**) and after resection of the tumor with cryopexy and 0.04% mitomycin C (MMC) application, followed by ocular surface re-construction by transplantation of free conjunctival graft from the same eye (**a**)

Primarily, treatment consisted of surgical excision of the tumor with sufficient safety margin, cryopexy in cases with broad expansion or unclear lesion boundaries, and intraoperative application of 0.04% mitomycin C (MMC) for 4 min (Fig. 4). In some cases, the lesions were reduced in size by preoperative instillation of 0.04% MMC eye drops. Postoperative instillation of 0.04% MMC or interferon (IFN)-α2b eye drops was used in advanced cases and recurrent lesions.

Among the 97 patients, 4 were treated at other hospitals. Of the remaining 93 patients, 9 (9.7%) received only 0.04% MMC or IFN-α2b instillation alone after incisional biopsy, and 84 patients (90.3%) underwent surgical excision with adjuvant therapy as described above. Re-construction after resection of the pathological conjunctiva was performed by transplantation of free bulbar conjunctiva, free tarsal plate with palpebral conjunctiva, or amniotic membrane, as needed. Three patients (3.2%) who had broad lesions at initial presentation were treated by EBRT alone. Seven patients (7.5%) required enucleation of the eyeball or orbital exenteration as the initial treatment.

Treatment outcome of 77 patients followed for 12 months or longer at our hospital were analyzed. The mean follow-up period was 35.6 ± 42.9 months. Local recurrence occurred in 24 patients (31.1%) and metastasis to regional lymph nodes was observed in 6 patients (6.2%). No metastasis to distant organs was found, and no patient died from SCC during the follow-up period.

### Melanoma (*n* = 60)

The mean age of patients diagnosed with melanoma was 65.8 ± 15.7 (range: 28–96) years. There were 32 men and 28 women, with no significant difference in proportion of men and women. No patient had bilateral lesion. Fifty patients (83.3%) had preexisting conjunctival melanosis.

**Fig. 5:**
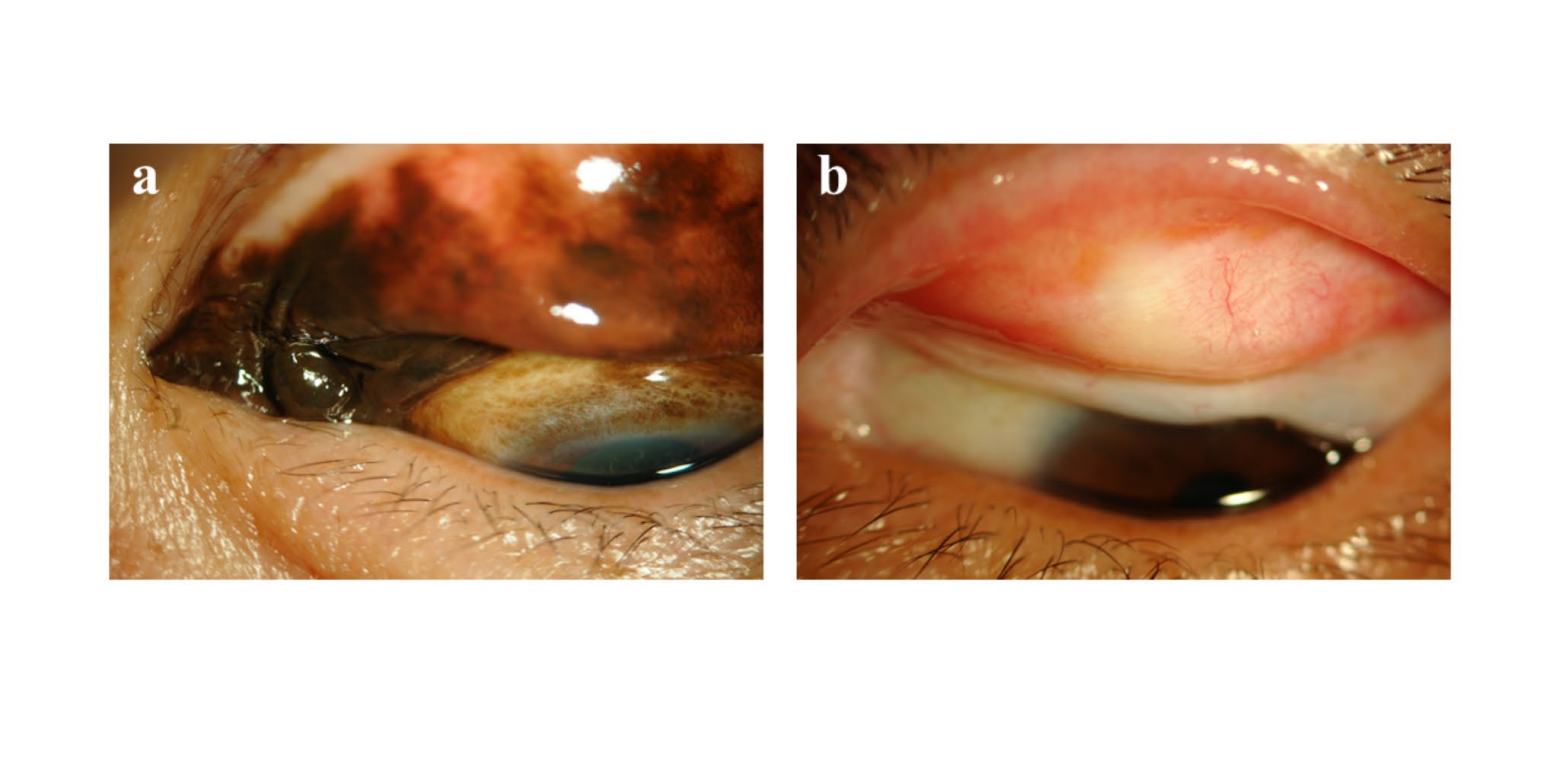
Melanoma: before treatment (**a**) and after resection of the tumor with carbon dioxide laser, cryopexy, and 0.04% MMC application (**b**). No recurrence and metastasis were observed for more than 3 years

Incisional biopsy for diagnostic purpose was not done for any case of melanoma. Surgical excision of the tumor with sufficient safety margin, cryopexy, and intraoperative 0.04% MMC application were performed as eye-preserving treatment. Carbon dioxide laser was also used in some eyes with palpebral conjunctival melanoma (Fig. 5). Postoperative instillation of 0.04% MMC or IFN-α2b eye drops was used in patients with broad lesions before treatment and patients who showed local recurrence. Orbital exenteration was performed as the initial treatment in 4 patients (6.7%) with broad lesions involving the eyelid and/or infraorbital invasion. Two patients who had advanced disease and several systemic disorders at the initial presentation did not receive any curative treatment, and opted for best supportive care.

Treatment outcomes of 45 patients who were followed for 24 months or longer at our hospital were analyzed. The mean follow-up period was 44.0 ± 21.2 months (24–105 months). Local recurrence was observed in 13 patients (28.9%) and metastases to regional lymph nodes or distant organs were confirmed in 13 patients (28.9%).　Seven patients (15.6%) died from tumor-related cause.

### Other tumors (*n* = 5)

**Fig. 6:**
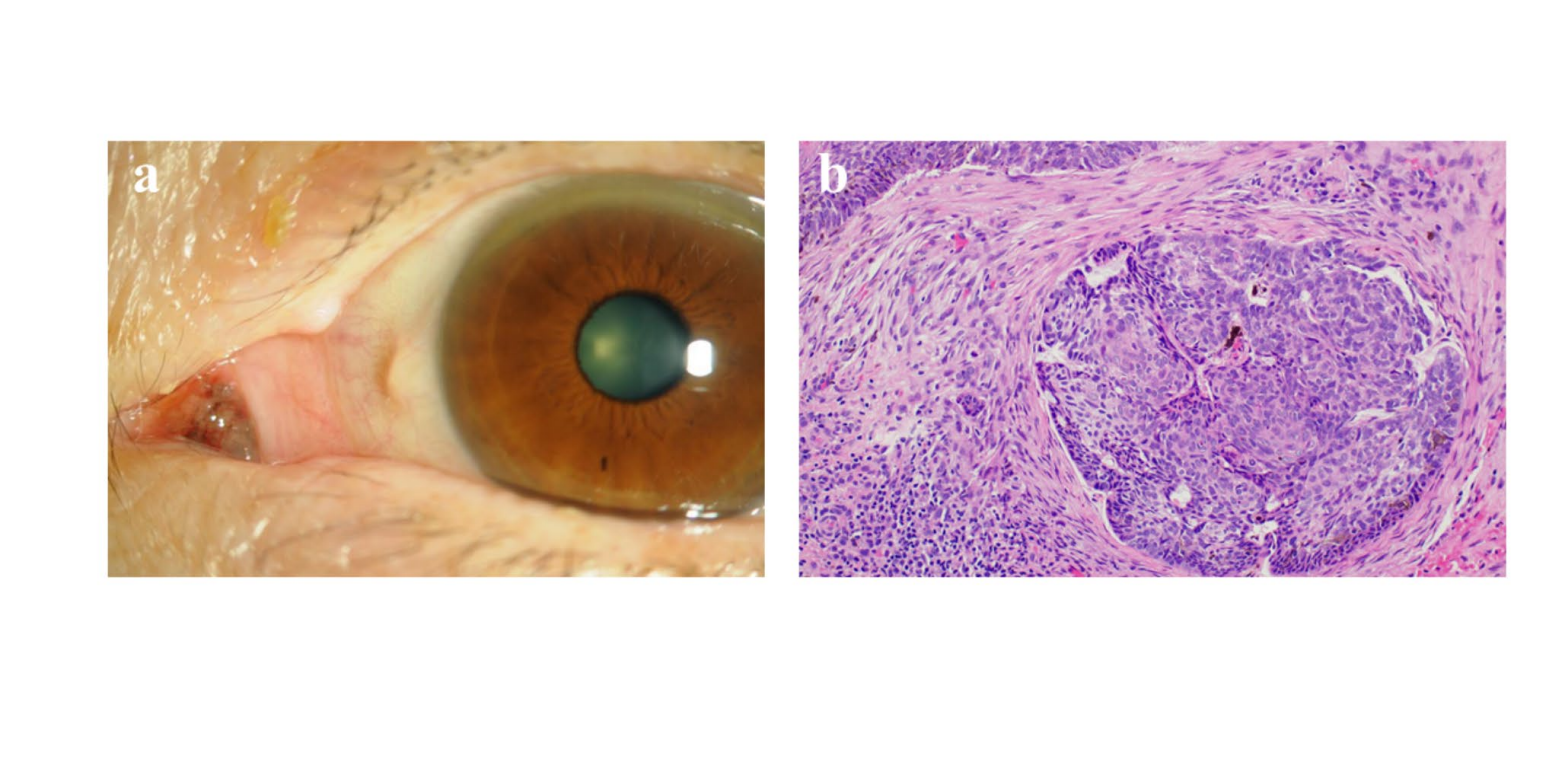
Basal cell carcinoma. Tiny, brown colored tumor arising from caruncle (**a**). Histopathology was compatible with basal cell carcinoma with palisading

Two patients with BCC (one man and one woman) were aged 76 years (Fig. 6) and 52 years, respectively. The BCC arose from the caruncle. One patient underwent simple resection of the caruncle lesion. Another patient underwent extended resection including the lacrimal sac due to invasive lesion [14], and this patient showed local recurrence 18 months after the initial treatment and required additional resection.

**Fig. 7:**
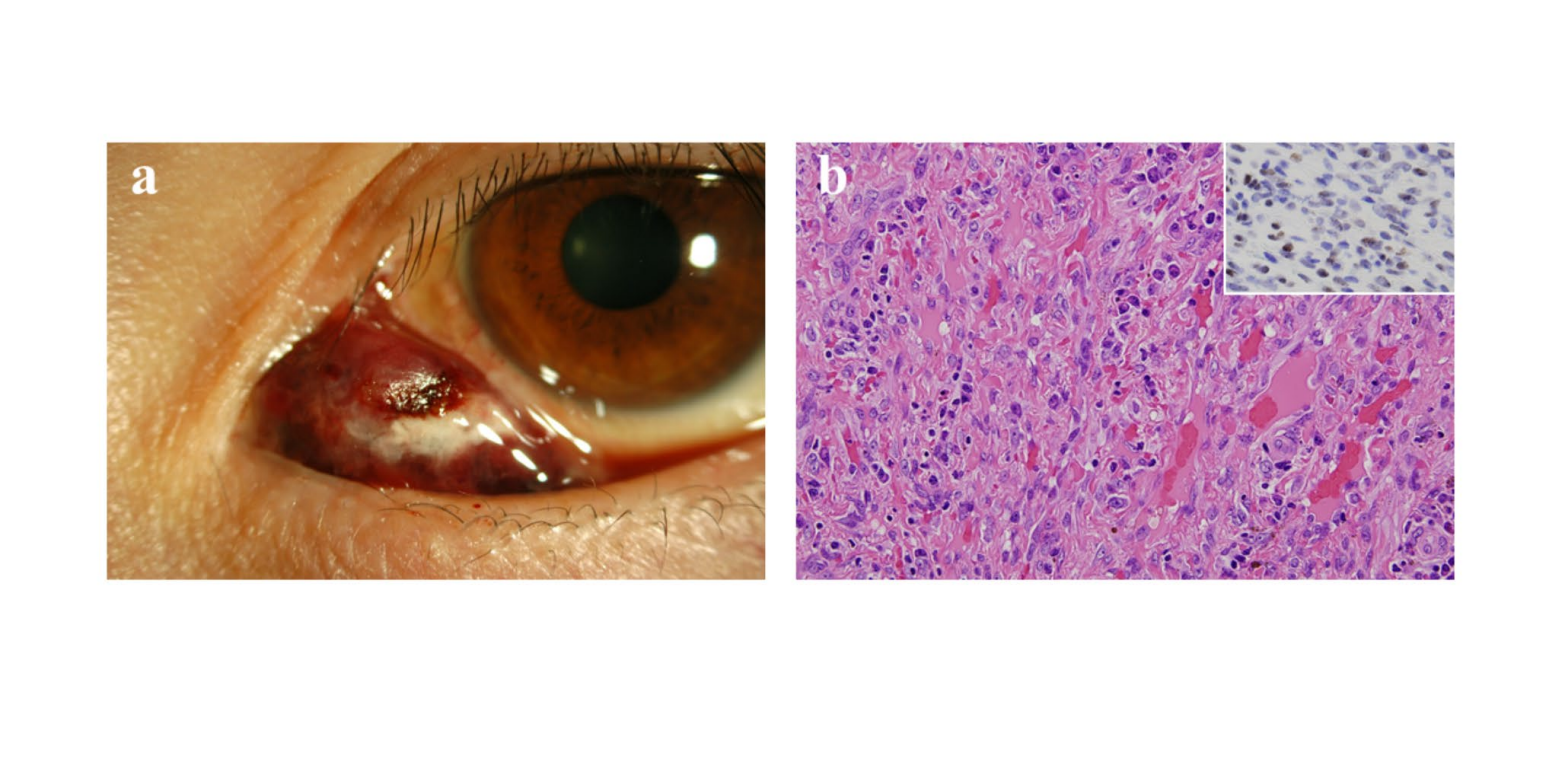
Kaposi’s sarcoma. Red, nodular lesion arising from palpebral conjunctiva (**a**). Characteristic histopathological findings with spindle cell proliferation and vascular clefts (**a**). Immunostaining is positive for HHV-8 (inset)

The two patients with Kaposi’s sarcoma were aged 44 years (Fig. 7) and 28 years. Both patients were men, and were positive for serum antihuman immunodeficiency virus (HIV) antibody. Both patients were treated with surgical resection, followed by systemic anti-retrovirus therapy and anti-human herpes virus (HHV)-8 therapy, with no recurrence for more than 12 months.

**Fig. 8:**
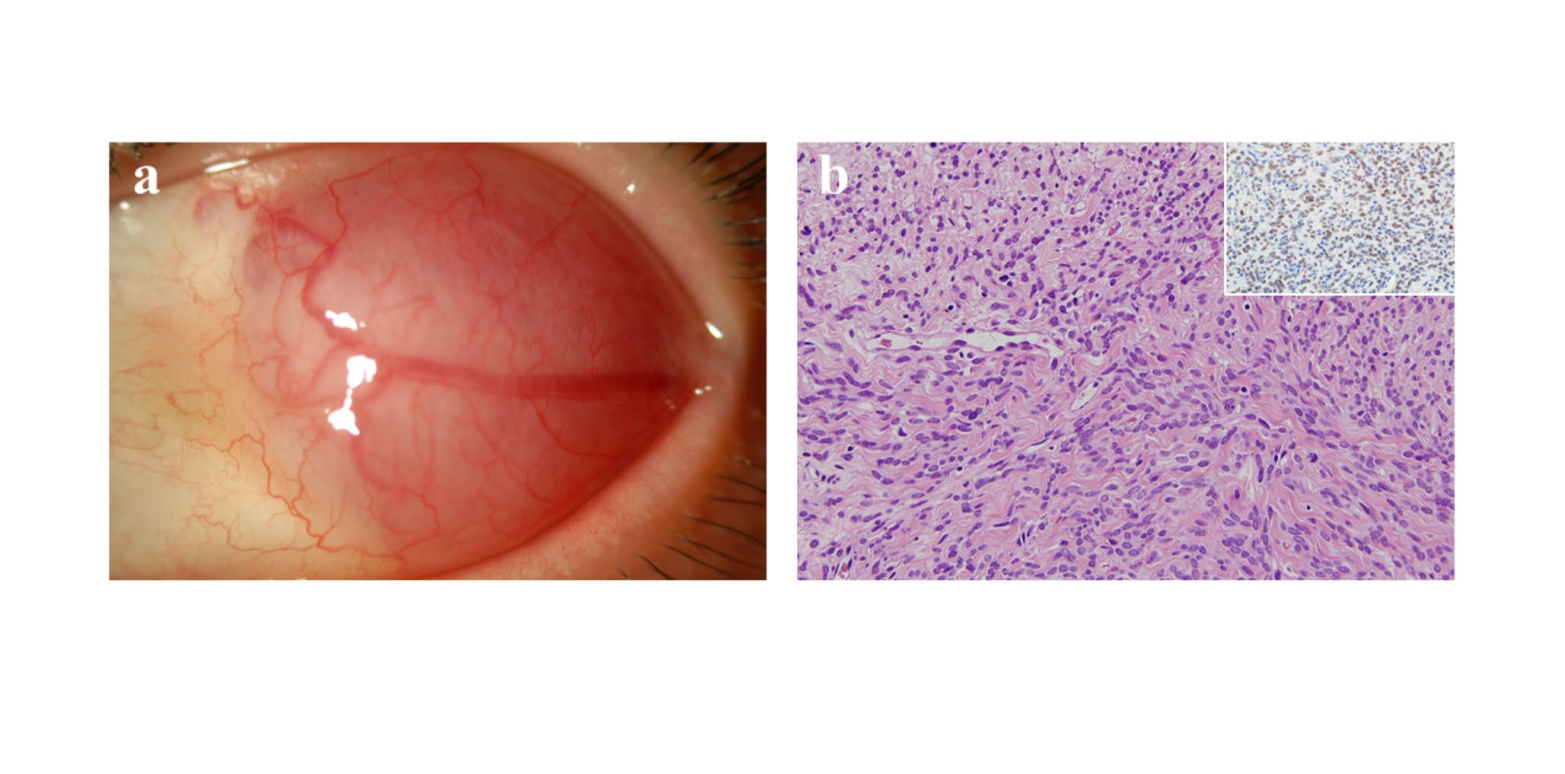
Solitary fibrous tumor. Red-pink, dome shaped lesion of the bulbar conjunctiva with dilated vessels (**a**). Spindle to ovoid cell proliferation surrounding blood vessels (**b**). Immunostaining is positive for STAT6 (inset)

The patient with SFT was a 35-year-old woman (Fig. 8). She was treated with simple excision of the conjunctival lesion, and no recurrence was observed during 24 months after surgery.

## Discussion

There are few reports that discuss the epidemiology of malignant conjunctival tumors, although a large number of articles referring to the clinical features and prognosis of OSSN, melanoma and lymphoma individually have been published. In the current series, lymphoma was the most common malignant conjunctival tumor (54.9%), followed by SCC (27.0%) and melanoma (16.7%). These results are quite different from reports from Western countries. In Europe and North America, OSSN including SCC has always been reported as the most common malignant conjunctival tumor [1, 15, 16]. In contrast, the present study and other reports from Asian countries [7, 17] show a higher incidence of lymphoma than in Europe and North America. The reason for the epidemiological differences is not clear. However, among malignant ocular adnexal tumors, there is a striking difference in the incidence of malignant eyelid tumors between East and West. In Europe and North America, basal cell carcinoma constitutes an overwhelmingly high percentage of malignant eyelid tumors, whereas the incidence of sebaceous carcinoma is very high in Asia including Japan [9–12]. The racial differences of patients with malignant ocular adnexal tumors, especially lymphoma of the conjunctiva and sebaceous carcinoma of the eyelid, may be associated with genetic or environmental factors [17]. These differences are an interesting issue that should be further investigated to elucidate the pathogenesis as well as the genetic factors involved in these malignant tumors.

We are planning to conduct a separate analysis of the treatment outcomes of conjunctival lymphoma and SCC according to various treatment modalities, with extension of the follow-up period. Detailed results will be reported in the future. Meanwhile, to summarize briefly the findings of the present study, the treatment outcomes of conjunctival lymphoma were extremely good regardless of the treatment modality. Local recurrence of lymphoma was observed in 8.4% after treatment in the present study. Kirkegaard et al. [18] report a recurrence rate of 37.9% (64 of 169) in patients with conjunctival lymphoma. Their study population was a large cohort from seven eye cancer centers, and EMZL, a low-grade B cell lymphoma, constituted 68.4%. Possible reasons for the favorable outcome in our current study may be the short follow-up period (minimum 12 months) and high proportion of EMZL of 89.3%.

In the present study, there was no significant difference in the recurrence rate of EMZL between the EBRT group (13.9%) and the surgical resection group (14.4%) (unpublished data). EBRT is often performed after incisional biopsy or even excisional biopsy. However, if there is no difference in recurrence rates even without radiotherapy, uniform EBRT for conjunctival EMZL may not be necessary, even though adverse events associated with low-dose EBRT rarely pose serious clinical problems. Of course, more aggressive types of lymphoma such as diffuse large B cell lymphoma require EBRT or systemic chemotherapy, and longer periods of observation including monitoring for extraocular lymphoma development should be considered.

Lee and Hirst [19] report that 30% of patients with conjunctival SCC recurred after surgical excision. Recently, many reports have demonstrated better outcomes for OSSN including SCC [1, 2, 20, 21]. This study focused on SCC, the recurrence rate was 31.1%, and most of the recurrence cases were cured by re-excision with or without local chemotherapy. The pathogenesis of OSSN is obscure, but ultraviolet exposure and human papilloma virus (HPV) have been suggested as related to the pathogenesis of OSSN, although HPV is not always detected [22]. Recent studies suggest that atopic dermatitis and allergic diseases are associated with the development of OSSN [23, 24]. The prevalence of atopic dermatitis in Japan is reported to be 2.1% [25]. In this study, 10 of 77 patients (13.0%) with SCC had a medical history of atopic dermatitis. This finding indicates a high prevalence of atopic dermatitis in patients with conjunctival SCC, and supports a previous study showing a relationship between atopic dermatitis and OSSN [23].

Conjunctival melanoma is an extremely rare ocular disease, especially in Asia including Japan [4, 26, 27]. Our report of 2023 is the only study with a relatively large number of cases (*n* = 50) from a single institute in Japan [28].

An epidemiologic analysis shows an increase in incidence of conjunctival melanoma in the United States, coinciding with the trend seen in cutaneous melanoma [5]. It is likely that a similar epidemiological change may also be observed in Asia and Japan, although precise data will be disclosed in the future.

In recent treatment of conjunctival melanoma, local chemotherapy with MMC and IFN-α2b has been universally performed along with surgical resection of the tumor, as in the treatment of conjunctival SCC [29, 30]. On the other hand, orbital exenteration remains a treatment option for advanced cases even today. We previously reported the treatments and outcomes of 45 patients with conjunctival melanoma followed for more than 12 months [28]. In the present study, although the local recurrence rate was the same as in the previous report, the number of metastasis cases increased from 7 (15.6%) to 13 cases (28.9%) due to extension of the follow-up period to 24 months or longer. There was a case in which local recurrence did not occur, but brain metastases developed 8 years after treatment. The present study suggests that conjunctival melanoma requires continuous follow-up for as long as possible, keeping in mind the possibility of metastasis to other organs.

BCC is known to arise from the conjunctiva, especially from the caruncle [14, 31]. Among the two cases in this study, one case had invasion to the lacrimal sac and recurred, but the outcome following surgery was good.

Kaposi’s sarcoma is an intermediate malignant soft tissue tumor. Conjunctival Kaposi’s sarcoma is a representative malignant tumor caused by HHV-8 especially associated with acquired immunodeficiency syndromes (AIDS) [32]. However, there are few reports of conjunctival Kaposi’s sarcoma at least in Japan.

SFT arising from the conjunctiva is extremely rare, with only a few case reports in the literature [33]. Although our case was clinically suspected to be a lymphoproliferative disease, it was diagnosed as SFT based on the characteristic histopathological findings including immunostaining.

This study has some limitations. Tumor laterality was determined when the histopathological diagnosis was made at our hospital. However, in cases of lymphoma and SCC, tumors can develop in the fellow eye during the course of the disease; therefore, the actual number of bilateral cases might be higher. Treatment outcomes including metastasis are affected by the follow-up period, as shown by the data from our melanoma cases. Obviously, similar effects may be observed in OSSN and lymphoma. Further study with extended follow-up period is needed. The present study was conducted in a single referral center in Japan. A population-based study would confirm the epidemiological trend of primary malignant conjunctival tumors in Japanese population.

Although the number of cases and length of follow-up were limited, we demonstrated the current epidemiology and treatment outcomes of conjunctival malignant tumors in Japan. As mentioned above, we plan to extend the follow-up period and analyze more cases and updated outcomes according to different treatment modalities.
